# Fuzzy Γ-Hyperideals in Γ-Hypersemirings by Using Triangular Norms

**DOI:** 10.1155/2014/428635

**Published:** 2014-05-20

**Authors:** B. A. Ersoy, B. Davvaz, S. Onar, V. Leoreanu-Fotea

**Affiliations:** ^1^Department of Mathematics, Yildiz Technical University, 81270 Istanbul, Turkey; ^2^Department of Mathematics, Yazd University, Yazd, Iran; ^3^Faculty of Mathematics, “Al.I. Cuza” University, Iasi, Romania

## Abstract

The concept of Γ-semihyperrings was introduced by Dehkordi and Davvaz as a generalization of semirings, semihyperrings, and Γ-semiring. In this paper, by using the notion of triangular norms, we define the concept of triangular fuzzy sub-Γ-semihyperrings as well as triangular fuzzy Γ-hyperideals of a Γ-semihyperring, and we study a few results in this respect.

## 1. Introduction

In [1], Nobusawa introduced Γ-rings as a generalization of ternary rings. Let *M* be an additive group whose elements are denoted by *a*, *b*, *c*,… and Γ another additive group whose elements are *γ*, *β*, *α*,…. Suppose that *aγb* is defined to be an element of *M* and that *γaβ* is defined to be an element of Γ for every *a*, *b*, *γ*, and *β*. If the products satisfy the following three conditions: (1)(*a*_1_ + *a*_2_)*γb* = *a*_1_ *γb* + *a*_2_ *γb*, *a*(*γ*_1_ + *γ*_2_)*b* = *aγ*_1_ *b* + *aγ*_2_ *b*, *aγ*(*b*_1_ + *b*_2_) = *aγb*_1_ + *aγb*_2_; (2)(*aγb*)*βc* = *aγ*(*b* *βc*) = *a*(*γb* *β*)*c*; (3) if *aγb* = 0 for any *a* and *b* in *M*, then *γ* = 0; then *M* is called a Γ-ring in the sense of Nobusawa [1]. Barnes [2] weakened slightly the conditions in the definition of Γ-ring and gave a new definition of a Γ-ring. Let *M* and Γ be two additive abelian groups. Suppose that there is a mapping from *M* × Γ × *M* → *M* (sending (*a*, *γ*, *b*) into *aγb* such that (1)  (*a*_1_ + *a*_2_)*γb* = *a*_1_ *γb* + *a*_2_ *γb*, *a*(*γ*_1_ + *γ*_2_)*b* = *aγ*_1_ *b* + *aγ*_2_ *b*, *aγ*(*b*_1_ + *b*_2_) = *aγb*_1_ + *aγb*_2_; (2)  (*aγb*)*βc* = *aγ*(*b* *βc*); then *M* is called a Γ-ring in the sense of Barnes [2]. Nowadays, Γ-rings mean the Γ-rings due to Barnes and other Γ-rings are known as Γ_*N*_-rings, that is, gamma rings in the sense of Nobusawa. Barnes [2], Luh [3], and Kyuno [4] studied the structure of Γ-rings and obtained various generalization analogous to corresponding parts in ring theory. The notion of Γ-semirings was introduced by Rao [5] as a generalization of semirings as well as Γ-rings. Subsequently, by introducing the notion of operator semirings of a Γ-semiring, Dutta and Sardar [6] enriched the theory of Γ-semirings. Algebraic hyperstructures represent a natural extension of classical algebraic structures and they were introduced by the French mathematician Marty [7]. Algebraic hyperstructures are a suitable generalization of classical algebraic structures. In a classical algebraic structure, the composition of two elements is an element, while in an algebraic structure, the composition of two elements is a set. Since then, hundreds of papers and several books have been written on this topic, for example, see [8–10]. In [11, 12], Dehkordi and Davvaz studied the notion of a Γ-semihyperring as a generalization of semiring, semihyperring, and Γ-semiring.

Fuzzy sets are sets whose elements have degrees of membership. Fuzzy sets have been introduced by Zadeh (1965) as an extension of the classical notion of sets [13]. Let *X* be a set. A fuzzy subset *A* of *X* is characterized by a membership function *μ*_*A*_ : *X* → [0,1] which associates with each point *x* ∈ *X* its grade or degree of membership *μ*_*μ*_(*x*) ∈ [0,1]. Fuzzy sets generalize classical sets since the characteristic functions of classical sets are special cases of the membership functions of fuzzy sets, if the latter only take values 0 or 1. After the introduction of fuzzy sets by Zadeh, reconsideration of the concept of classical mathematics began. In 1971, Rosenfeld [14] introduced fuzzy sets in the context of group theory and formulated the concept of a fuzzy subgroup of a group. Das characterized fuzzy subgroups by their level of subgroups in [15], since then many notions of fuzzy group theory can be equivalently characterized with the help of notion of level subgroups. The concept of a fuzzy ideal of a ring was introduced by Liu [16]. In 1992, Jun and Lee [17] introduced the notion of fuzzy ideals in Γ-rings and studied a few properties. In [6], Dutta and Sardar studied the structures of fuzzy ideals of Γ-rings. Also, see [18]. The study of fuzzy hyperstructures is an interesting research topic of fuzzy sets. There is a considerable amount of work on the connections between fuzzy sets and hyperstructures. In [19], Davvaz introduced the concept of fuzzy *H*_*v*_-ideals of *H*_*v*_-rings. Then, this concept was studied in depth in several papers, for example, see [20]. Also, see [21]. In [22, 23], Ersoy and Davvaz investigated some properties of fuzzy Γ-hyperideals of Γ-semihyperring. Now, in this paper, we define the concept of triangular fuzzy sub-Γ-semihyperrings and fuzzy Γ-hyperideals of a Γ-semihyperring by using triangular norms, and we study a few results in this respect.

## 2. Basic Concepts

Let *H* be a nonempty set and let ℘*(*H*) be the set of all nonempty subsets of *H*. A hyperoperation on *H* is a map ∘:*H* × *H* → ℘*(*H*) and the couple (*H*, ∘) is called a hypergroupoid. If *A* and *B* are nonempty subsets of *H*, then we denote *A*∘*B* = ⋃_*a*∈*A*,*b*∈*B*_ *a*∘*b*, *x*∘*A* = {*x*}∘*A* and *A*∘*x* = *A*∘{*x*}. A hypergroupoid (*H*, ∘) is called a semihypergroup if for all *x*, *y*, *z* of *H* we have (*x*∘*y*)∘*z* = *x*∘(*y*∘*z*), which means that ⋃_*u*∈*x*∘*y*_ *u*∘*z* = ⋃_*v*∈*y*∘*z*_ *x*∘*v*. A semihyperring is an algebraic structure (*R*, +, ·) which satisfies the following properties: (1)  (*R*, +) is a commutative semihypergroup; that is, (*x* + *y*) + *z* = *x* + (*y* + *z*) and *x* + *y* = *y* + *x* for all *x*, *y*, *z* ∈ *R*; (2)  (*R*, ·) is a semihypergroup; (3) the hyperoperation · is distributive with respect to the hyperoperation +; that is, *x* · (*y* + *z*) = *x* · *y* + *x* · *z*, (*x* + *y*) · *z* = *x* · *z* + *y* · *z* for all *x*, *y*, *z* ∈ *R*; (4) the element 0 ∈ *R* is an absorbing element; that is *x* · 0 = 0 · *x* = 0 for all *x* ∈ *R*. A semihyperring (*R*, +, ·) is called commutative if and only if *a* · *b* = *b* · *a* for all *a*, *b* ∈ *R*. Vougiouklis in [24] studied the notion of semihyperrings in a general form; that is, both the sum and product are hyperoperations; also see [25]. A semihyperring (*R*, +, ·) with identity 1_*R*_ ∈ *R* means that 1_*R*_ · *x* = *x* · 1_*R*_ = *x* for all *x* ∈ *R*. The concept of Γ-semihyperring is introduced and studied by Dehkordi and Davvaz [11]. Let *R* be a commutative semihypergroup and Γ be a commutative group. Then, *R* is called a Γ-semihyperring if there exists a map *R* × Γ × *R* → ℘*(*R*) (image to be denoted by *aαb* for *a*, *b* ∈ *R* and *α* ∈ Γ) and ℘*(*R*) is the set of all nonempty subsets of *R* satisfying the following conditions:

1. *aα*(*b* + *c*) = *aαb* + *aα* *c*,
2. (*a* + *b*)*αc* = *aα* *c* + *b* *αc*,
3. *a*(*α* + *β*)*b* = *aαb* + *aβb*,
4. *aα*(*b* *βc*) = (*aαb*)*βc*,

for all *a*, *b*, *c* ∈ *R* and for all *α*, *β* ∈ Γ. One can find many examples of Γ-semihyperrings in [11, 12]. In the above definition if *R* is a semigroup, then *R* is called a multiplicative Γ-semihyperring. A Γ-semihyperring *R* is called commutative if *xαy* = *yαx* for every *x*, *y* ∈ *R* and *α* ∈ Γ. We say that Γ-semihyperring *R* with zero if there exists 0 ∈ *R* such that *a* ∈ *a* + 0, 0 ∈ 0*αa*, 0 ∈ *aα*0 for all *a* ∈ *R* and *α* ∈ Γ. Let *A* and *B* be two nonempty subsets of Γ-semihyperring *R* and *x* ∈ *R*. We define *A* + *B* = {*x* | *x* ∈ *a* + *b*; *a* ∈ *A*, *b* ∈ *B*} and *A*Γ*B* = {*x* | *x* ∈ *aαb*; *a* ∈ *A*, *b* ∈ *B*, *α* ∈ Γ}. A nonempty subset *R*_1_ of Γ-semihyperring *R* is called a sub-Γ-semihyperring if it is closed with respect to the multiplication and addition. In other words, a nonempty subset *R*_1_ of Γ-semihyperring *R* is a sub-Γ-semihypergroup if *R*_1_ + *R*_1_⊆*R*_1_ and *R*_1_Γ*R*_1_⊆*R*_1_.

A right (left) Γ-hyperideal *I* of a Γ-semihyperring *R* is an additive sub-semihypergroup (*R*, +) such that *I*Γ*R*⊆*I* (*R*Γ*I*⊆*I*). If *I* is both right and left Γ-hyperideal of *R*, then we say that *I* is a two-sided Γ-hyperideal or simply a Γ-hyperideal of *R*. In [22, 23], Ersoy and Davvaz studied fuzzy Γ-hyperideals of Γ-semihyperrings. We recall the notion of a fuzzy Γ-hyperideal of a Γ-semihyperring. Let *R* be a Γ-semihyperring and *μ* be a fuzzy subset of *R*. Then (1)  *μ* is called a fuzzy left Γ-hyperideal of *R* if min⁡⁡{*μ*(*x*), *μ*(*y*)} ≤ inf⁡_*z*∈*x*+*y*_{*μ*(*z*)} and *μ*(*y*) ≤ inf⁡_*z*∈*xγy*_{*μ*(*z*)} for all *x*, *y* ∈ *R* and *γ* ∈ Γ; (2)  *μ* is called a fuzzy right Γ-hyperideal of *R* if min⁡⁡{*μ*(*x*), *μ*(*y*)} ≤ inf⁡_*z*∈*x*+*y*_{*μ*(*z*)} and *μ*(*x*) ≤ inf⁡_*z*∈*xγy*_{*μ*(*z*)} for all *x*, *y* ∈ *R* and *γ* ∈ Γ; (3)  *μ* is called a fuzzy Γ-hyperideal of *R* if it is both a fuzzy left Γ-hyperideal and a fuzzy right Γ-hyperideal of *R*.

The concept of a triangular norm was introduced by Menger [26] in order to generalize the triangular inequality of a metric. The current notion of a* t*-norm and its dual operation is due to Schweizer and Sklar [27]. By a* t*-norm *T* we mean a function *T* : [0,1] × [0,1] → [0,1] satisfying the following conditions: (T1) *T*(*x*, *y*) = *T*(*y*, *x*); (T2) *T*(*x*, *T*(*y*, *z*)) = *T*(*T*(*x*, *y*), *z*); (T3) *T*(*x*, *y*) ≤ *T*(*x*, *z*) if *y* ≤ *z*; (T4) *T*(*x*, 1) = *x*, for all *x*, *y*, *z* ∈ [0,1]. For every* t*-norm *T*, we set Δ_*T*_ = {*x* ∈ [0,1], *T*(*x*, *x*) = *x*}. A* t*-norm on [0,1] is called a continuous* t*-norm if *T* is a continuous function from [0,1] × [0,1] to [0,1] with respect to the usual topology. Note that the function “Min” is a continuous* t*-norm. A triangular conorm (*t*-norm for short) is a binary operation *S* on the unit interval [0,1], that is, a function *S* : [0,1]×[0,1]→[0,1], which for all *x*, *y*, *z* ∈ [0,1] satisfies (T1)–(T3) and (S4) *S*(*x*, 0) = *x*. From an axiomatic point of view,* t*-norms and* t*-conorms differ only with respect to their respective boundary conditions. In fact, the concepts of* t*-norms and* t*-conorms are dual in some sense. Anthony and Sherwood [28] redefined a fuzzy subgroup of a group by using the notion of* t*-norm.

## 3. *T*-Fuzzy Sub-Γ-Semihyperrings and *T*-Fuzzy Γ-Hyperideals

In this section, we define the notion of *T*-fuzzy sub-Γ-semihyperrings and *T*-fuzzy Γ-hyperideals of a Γ-semihyperring and we study some of their properties. Let *T* be a* t*-norm and *μ* be a fuzzy subset of a Γ-semihyperring *R*. Then, we say *μ* has imaginable property if *Im*⁡*μ*⊆Δ_*T*_.

**Definition 1**

Let *R* be a Γ-semihyperring, *T* be a *t*-norm, and *μ* be a fuzzy subset of *R*. Then, *μ* is called a *T*-fuzzy sub-Γ-semihyperring of *R* if

1. *T*(*μ*(*x*), *μ*(*y*)) ≤ inf⁡_*z*∈*x*+*y*_{*μ*(*z*)},
2. *T*(*μ*(*x*), *μ*(*y*)) ≤ inf⁡_*z*∈*xγy*_{*μ*(*z*)},

for all *x*, *y* ∈ *R* and for all *γ* ∈ Γ.

A *T*-fuzzy sub-Γ-semihyperring of *R* is said to be imaginable if it satisfies the imaginable property. Clearly, if *R* is a Γ-semiring, then *μ* is a *T*-fuzzy sub-Γ-semiring of *R* when

 (1′) *T*(*μ*(*x*), *μ*(*y*)) ≤ *μ*(*x* + *y*),
 (2′) *T*(*μ*(*x*), *μ*(*y*)) ≤ *μ*(*xγy*),

for all *x*, *y* ∈ *R* and for all *γ* ∈ Γ.

**Example 2**

Suppose that *R* = *N*, the set of natural numbers, and Γ is a nonempty subset of *R*. For any *x*, *y* ∈ *R* and *γ* ∈ Γ, we define *x* + *y* = {*x*, *y*}  and *xγy* = {*x*, *γ*, *y*}. Then, *R* is a Γ-semihyperring. We define the fuzzy subset *μ* of *R* by

(1) $$\begin{array}{c} \mu \left(x\right)=\{\begin{array}{cc} \frac{3}{4} & \text{if}\;\;x\in \Gamma \\ \frac{5}{9} & \text{otherwise} \end{array} \end{array}$$

and we consider the *t*-norm *T*(*r*, *s*) = *rs*/(2 − (*r* + *s* − *rs*)), where *r*, *s* ∈ [0,1]. Then, for any *x*, *y* ∈ *R* and *γ* ∈ Γ, we have

(2) inf⁡z∈x+y{μ(z)}=min⁡{μ(x),μ(y)}={34if  x,y∈Γ59otherwise,inf⁡z∈xγy{μ(z)}=min⁡{μ(x),μ(γ),μ(y)}=59.

On the other hand, we have the following cases:

1. *x*, *y* ∈ Γ,
2. *x* ∉ Γ and *y* ∈ Γ (or, *x* ∈ Γ  and  *y* ∉ Γ),
3. *x*, *y* ∉ Γ.

Regarding the above cases, we have *T*(3/4, 3/4) = 9/17, *T*(3/4, 5/9) = 15/40, and *T*(5/9, 5/9) = 25/81. Thus, in every case, we obtain

(3) $$\begin{array}{c} T\left(\mu \left(x\right),\mu \left(y\right)\right)\leq \underset{z\in x+y}{\inf}\left\{\mu \left(z\right)\right\},\\ T\left(\mu \left(x\right),\mu \left(y\right)\right)\leq \underset{z\in x\gamma y}{\inf}\left\{\mu \left(z\right)\right\}. \end{array}$$

Therefore, *μ* is a *T*-fuzzy sub-Γ-semihyperring of *R*.

**Lemma 3**

Let *R* be a Γ-semihyperring, *T* be a *t*-norm, and *μ* be a *T*-fuzzy sub-Γ-semihyperring of *S*. Then

(4) $$\begin{array}{c} T_{n}\left(\mu \left(x_{1}\right),\ldots ,\mu \left(x_{n}\right)\right)\leq \underset{z\in x_{1}+\cdots +x_{n}}{\inf}\left\{\mu \left(z\right)\right\},\\ T_{n}\left(\mu \left(x_{1}\right),\ldots ,\mu \left(x_{n}\right)\right)\leq \underset{z\in x_{1}\gamma \cdots \gamma x_{n}}{\inf}\left\{\mu \left(z\right)\right\}, \end{array}$$

for all *x*_1_,…, *x*_*n*_ ∈ *R* and *γ* ∈ Γ, where

(5) Tn(t1,…,tn) ={t1if  n=1T(t1,t2)if  n=2T(ti,Tn−1(t1,…,ti−1,ti+1,…,tn))if  n>2.

**Proof**

The proof is straightforward by mathematical induction.

**Lemma 4**

Let *R* be a Γ-semihyperring, *T* be a *t*-norm, and *μ* be a *T*-fuzzy sub-Γ-semihyperring of *S*. Let *A* and *B* be nonempty subsets of *R*. Then

(6) $$\begin{array}{c} T\left(\underset{a\in A}{\inf}\left\{\mu \left(a\right)\right\},\underset{b\in B}{\inf}\left\{\mu \left(b\right)\right\}\right)\leq \underset{z\in A+B}{\inf}\left\{\mu \left(z\right)\right\},\\ T\left(\underset{a\in A}{\inf}\left\{\mu \left(a\right)\right\},\underset{b\in B}{\inf}\left\{\mu \left(b\right)\right\}\right)\leq \underset{z\in A\gamma B}{\inf}\left\{\mu \left(z\right)\right\}, \end{array}$$

for all *γ* ∈ Γ.

**Proof**

The proof is straightforward.

**Theorem 5**

Let *R* be a Γ-semihyperring, *T* be a *t*-norm, and *μ* be a fuzzy subset of *S* with imaginable property and *b* the maximum of Im *μ*. Then, the following two statements are equivalent:

1. *μ* is a *T*-fuzzy sub-Γ-semihyperring of *S*,
2. *μ* ^−1^[*a*, *b*] is a sub-Γ-semihyperring of *S* whenever *a* ∈ Δ_*T*_ and 0 < *a* ≤ *b*.

**Proof**

(1)⇒(2): Suppose that *a* ∈ Δ_*T*_ and 0 < *a* ≤ *b*. If *x*, *y* ∈ *μ* ^−1^[*a*, *b*], then inf⁡_*z*∈*x*+*y*_⁡{*μ*(*z*)} ≥ *T*(*μ*(*x*), *μ*(*y*)) ≥ *T*(*a*, *a*) = *a*, which implies that *x* + *y*⊆*μ* ^−1^[*a*, *b*]. Similarly, assume that *a* ∈ Δ_*T*_ and 0 < *a* ≤ *b*. If *x*, *y* ∈ *μ* ^−1^[*a*, *b*] and *γ* ∈ Γ, then inf⁡_*z*∈*xγy*_⁡{*μ*(*z*)} ≥ *T*(*μ*(*x*), *μ*(*y*)) ≥ *T*(*a*, *a*) = *a*. Then, we have *xγy*⊆*μ* ^−1^[*a*, *b*], and so *μ* ^−1^[*a*, *b*] is a sub-Γ-semihyperring of *R*.

(2)⇒(1): Suppose that *x*, *y* ∈ *S* and *γ* ∈ Γ. Since *Im* *μ*⊆Δ_*T*_, both *μ*(*x*) and *μ*(*y*) are in Δ_*T*_. Now, we have

(7) T(T(μ(x),μ(y)),T(μ(x),μ(y))) =T(T(μ(x),T(μ(y),μ(x))),μ(y)) =T(T(μ(x),T(μ(x),μ(y))),μ(y)) =T(T(μ(x),μ(x)),T(μ(y),μ(y))) =T(μ(x),μ(y)),

and so *T*(*μ*(*x*), *μ*(*y*)) ∈ Δ_*T*_. Assume that *a* = *T*(*μ*(*x*), *μ*(*y*)). If *a* = 0, then

(8) $$\begin{array}{c} T\left(\mu \left(x\right),\mu \left(y\right)\right)=0\leq \underset{z\in x+y}{\inf}\left\{\mu \left(z\right)\right\},\\ T\left(\mu \left(x\right),\mu \left(y\right)\right)=0\leq \underset{z\in x\gamma y}{\inf}\left\{\mu \left(z\right)\right\}. \end{array}$$

Now, let 0 < *a* = *T*(*μ*(*x*), *μ*(*y*)) ≤ *μ*(*x*)∧*μ*(*y*) ≤ *μ*(*x*) ≤ *b*. Hence *x*, *y* ∈ *μ* ^−1^[*a*, *b*], which implies that *x* + *y*⊆*μ* ^−1^[*a*, *b*] and *xγy*⊆*μ* ^−1^[*a*, *b*]. Therefore *T*(*μ*(*x*), *μ*(*y*)) ≤ inf⁡_*z*∈*x*+*y*_⁡{*μ*(*z*)} and *T*(*μ*(*x*), *μ*(*y*)) ≤ inf⁡_*z*∈*xγy*_⁡{*μ*(*z*)}.

**Definition 6**

Let *R* be a Γ-semihyperring, *T* be a *t*-norm, and *μ* be a fuzzy subset of *R*. Then

(1) *μ* is called a *T*-fuzzy left Γ-hyperideal of *R* if
  (9) $$\begin{array}{c} T\left(\mu \left(x\right),\mu \left(y\right)\right)\leq \underset{z\in x+y}{\inf}\left\{\mu \left(z\right)\right\},\;\forall x,y\in R,\\ \mu \left(y\right)\leq \underset{z\in x\gamma y}{\inf}\left\{\mu \left(z\right)\right\},\;\forall x,y\in R,\;\;\forall \gamma \in \Gamma . \end{array}$$
(2) *μ* is called a *T*-fuzzy right Γ-hyperideal of *R* if
  (10) $$\begin{array}{c} T\left(\mu \left(x\right),\mu \left(y\right)\right)\leq \underset{z\in x+y}{\inf}\left\{\mu \left(z\right)\right\},\;\forall x,y\in R,\\ \mu \left(x\right)\leq \underset{z\in x\gamma y}{\inf}\left\{\mu \left(z\right)\right\},\;\forall x,y\in R,\;\;\forall \gamma \in \Gamma . \end{array}$$
(3) *μ* is called a *T*-fuzzy Γ-hyperideal of *R* if it is both a *T*-fuzzy left Γ-hyperideal and a *T*-fuzzy right Γ-hyperideal of *R*.

**Theorem 7**

Let *R* be a Γ-semihyperring, *T* be a *t*-norm, and *μ* be a fuzzy subset of *S* with imaginable property and *b* the maximum of Im *μ*. Then, the following two statements are equivalent:

1. *μ* is a *T*-fuzzy Γ-hyperideal of *R*,
2. *μ* ^−1^[*a*, *b*] is a Γ-hyperideal of *R* whenever *a* ∈ Δ_*T*_ and 0 < *a* ≤ *b*.

**Proof**

The proof is similar to the proof of Theorem 5.

Let *μ* be a fuzzy subset of *R* and *t* ∈ [0,1]. The set *U*(*μ*; *t*) = {*x* ∈ *R* | *μ*(*x*) ≥ *t*} is called a level subset of *μ*. So, we obtain the following corollary.

**Corollary 8**

Let *R* be a Γ-semihyperring and *μ* be a fuzzy subset of *R*. Then

1. *μ* is a Min-fuzzy sub-Γ-semihyperring of *R* if and only if every nonempty level subset is a sub-Γ-semihyperring of *R*;
2. *μ* is a Min-fuzzy Γ-hyperideal of *R* if and only if every nonempty level subset is a Γ-hyperideal of *R*.

**Corollary 9**

Let *A* be a subset of *R*. Then

1. the characteristic function *χ*_*A*_ is a *T*-fuzzy sub-Γ-semihyperring of *R* if and only if *A* is a sub-Γ-semihyperring of *R*;
2. the characteristic function *χ*_*A*_ is a *T*-fuzzy Γ-hyperideal of *R* if and only if *A* is a Γ-hyperideal of *R*.

**Theorem 10**

Let *R* be a Γ-semihyperring and *K* be a sub-Γ-semihyperring of *R*. Let *T* be the *t*-norm defined by *T*(*a*, *b*) = max⁡⁡{0, *a* + *b* − 1} and *μ* be a fuzzy subset of *R* defined by

(11) $$\begin{array}{c} \mu \left(x\right)=\{\begin{array}{cc} r & if\;\;x\in K\\ s & otherwise, \end{array} \end{array}$$

for all *a*, *b* ∈ [0,1] and *x* ∈ *R*, where *r*, *s* ∈ [0,1] such that *s* < *r*. Then, *μ* is a *T*-fuzzy sub-Γ-semihyperring of *R*. In particular, if *r* = 1 and *s* = 0, then *μ* is imaginable.

**Proof**

The proof is similar to the proof of Theorem  2.6 in [29].

**Definition 11**

Let *R*_1_ and *R*_2_ be Γ_1_ and Γ_2_-semihyperrings, respectively. If there exists a map *φ* : *R*_1_ → *R*_2_ and a bijection *f* : Γ_1_ → Γ_2_ such that

(12) $$\begin{array}{c} \varphi \left(x+y\right)=\left\{\varphi \left(z\right)\;|\;z\in x+y\right\}=\varphi \left(x\right)+\varphi \left(y\right),\\ \varphi \left(x\gamma y\right)=\left\{\varphi \left(z\right)\;|\;z\in x\gamma y\right\}=\varphi \left(x\right)f\left(\gamma \right)\varphi \left(y\right), \end{array}$$

for all *x*, *y* ∈ *R*_1_ and *γ* ∈ Γ, then we say (*φ*, *f*) is a homomorphism from *R*_1_ to *R*_2_. Also, if *φ* is a bijection then (*φ*, *f*) is called an isomorphism and *R*_1_ and *R*_2_ are isomorphic.

**Proposition 12**

Let *R*_1_ and *R*_2_ be Γ_1_ and Γ_2_-semihyperrings, respectively. Let (*φ*, *f*) be a homomorphism from *R*_1_ to *R*_2_. If *λ* is a *T*-fuzzy sub-Γ-semihyperring of *R*_2_, then *φ* ^−1^(*λ*) is a *T*-fuzzy sub-Γ-semihyperring of *R*_1_ too.

**Proof**

Suppose that *x*, *y* ∈ *R*_1_ and *γ* ∈ Γ. Then, we have

(13) inf⁡z∈x+y{φ−1(λ)(z)}=inf⁡z∈x+y{λ(φ(z))}≥inf⁡φ(z)∈φ(x+y){λ(φ(z))}≥inf⁡φ(z)∈φ(x)+φ(y){λ(φ(z))}≥T(λ(φ(x)),λ(φ(y)))=T(φ−1(λ)(x),φ−1(λ)(y));inf⁡z∈xγy{φ−1(λ)(z)}=inf⁡z∈xγy{λ(φ(z))}≥inf⁡φ(z)∈φ(xγy){λ(φ(z))}≥inf⁡φ(z)∈φ(x)f(γ)φ(y){λ(φ(z))}≥T(λ(φ(x)),λ(φ(y)))=T(φ−1(λ)(x),φ−1(λ)(y)).

Therefore, *φ* ^−1^(*λ*) is a *T*-fuzzy sub-Γ-semihyperring of *R*_1_.

**Proposition 13**

Let *R*_1_ and *R*_2_ be Γ_1_ and Γ_2_-semihyperrings, respectively. Let (*φ*, *f*) be a homomorphism from *R*_1_ to *R*_2_. If *λ* is a *T*-fuzzy Γ-hyperideal of *R*_2_, then *φ* ^−1^(*λ*) is a *T*-fuzzy Γ-hyperideal of *R*_1_ too.

**Proof**

The proof is similar to the proof of Proposition 12.

Let {*a*_*i*_}_*i*∈*I*_ and {*b*_*j*_}_*j*∈*J*_ be two sets of real numbers in [0,1]. Then, we say *T* is infinitely distributive if

(14) T(sup⁡i∈I{ai},sup⁡j∈J{bj})=sup⁡i∈Ij∈J⁡{T(ai,bj)}.

If *T* is continuous, then *T* is infinitely distributive [30].

**Lemma 14**

Let *T* be a continuous *t*-norm and {*μ*_*i*_}_*i*∈*I*_ be a family of *T*-fuzzy sub-Γ-semihyperring of *R*. Then, ⋂_*i*∈*I*_ *μ*_*i*_ is a *T*-fuzzy sub-Γ-semihyperring of *R*.

**Proof**

For any *x*, *y* ∈ *R* and *γ* ∈ Γ, we have

(15) inf⁡z∈x+y{(⋂i∈Iμi)(z)}=inf⁡z∈x+y{inf⁡i∈I{μi(z)}}=inf⁡i∈I{inf⁡z∈x+y{μi(z)}}≥inf⁡i∈I{T(μi(x),μi(y))}=T(inf⁡i∈I{μi(x)},inf⁡i∈I{μi(y)})=T((⋂i∈Iμi)(x),(⋂i∈Iμi)(y));inf⁡z∈xγy{(⋂i∈Iμi)(z)}=inf⁡z∈xγy{inf⁡i∈I{μi(z)}}=inf⁡i∈I{inf⁡z∈xγy{μi(z)}}≥inf⁡i∈I{T(μi(x),μi(y))}=T(inf⁡i∈I{μi(x)},inf⁡i∈I{μi(y)})=T((⋂i∈Iμi)(x),(⋂i∈Iμi)(y)).

**Lemma 15**

Let *R*_1_ and *R*_2_ be Γ_1_ and Γ_2_-semihyperrings, respectively, and (*φ*, *f*) be an onto homomorphism from *R*_1_ to *R*_2_. Then, for every *t* ∈ [0,1], we have *φ*(*μ*)_*t*_ = ⋂_*t*>*ϵ*>0_ *φ*(*μ*_*t*−*ϵ*_).

**Proof**

The proof is similar to the proof of Lemma  3.5 in [31].

**Proposition 16**

Let *R*_1_ and *R*_2_ be Γ_1_ and Γ_2_-semihyperrings, respectively, and let *μ* be a fuzzy subset of *R*_1_. Let (*φ*, *f*) be an onto homomorphism from *R*_1_ to *R*_2_. If *μ* is a Min-fuzzy sub-Γ-semihyperring of *R*_1_, then *φ*(*μ*) is a Min-fuzzy sub-Γ-semihyperring of *R*_2_ too.

**Proof**

Suppose that *μ* is a Max-fuzzy sub-Γ-semihyperring of *R*_1_. By Corollary 8, *φ*(*μ*) is a Max-fuzzy sub-Γ-semihyperring of *R*_2_ if every nonempty level subset *U*(*φ*((*μ*); *t*)) is a sub-Γ-semihyperring of *R*_2_. Thus, assume that *U*(*φ*((*μ*); *t*)) is any nonempty level subset. If *t* = 0, then *U*(*φ*((*μ*); *t*)) = *R*_2_, and if 0 < *t* ≤ 1, then by Lemma 15, we have *U*(*φ*((*μ*); *t*)) = ⋂_*t*>*ϵ*>0_ *φ*(*U*(*μ*; *t* − *ϵ*)). By Corollary 8, *U*(*μ*; *t* − *ϵ*) for each *t* > *ϵ* > 0 is a sub-Γ-semihyperring of *R*_1_. Hence, *φ*(*U*(*μ*; *t* − *ϵ*)) is a sub-Γ-semihyperring of *R*_2_. By Lemma 14, *φ*(*U*(*μ*; *t*)) being an intersection of a family of sub-Γ-semihyperrings is also a sub-Γ-semihyperring of *R*_2_ and the proof is completed.

**Definition 17**

Let *R*_1_ and *R*_2_ be two Γ-semihyperrings and let *μ* and *λ* be fuzzy subsets of *R*_1_ and *R*_2_, respectively. The product of *μ* and *λ* is defined to be the fuzzy subset *μ* × *λ* of *R*_1_ × *R*_2_ with (*μ* × *λ*)(*x*, *y*) = *T*(*μ*(*x*), *λ*(*x*)), for all (*x*, *y*) ∈ *R*_1_ × *R*_2_.

**Proposition 18**

Let *R*_1_ and *R*_2_ be two Γ-semihyperrings and let *μ* and *λ* be fuzzy subsets of *R*_1_ and *R*_2_, respectively. Then

1. *if μ and λ are T-fuzzy sub-*Γ*-semihyperrings of R*_1_ *and R*_2_ *, respectively, then μ* × *λ is a T-fuzzy sub-*Γ*-semihyperring of R*_1_ × *R*_2_ *; *
2. *if μ and λ are T-fuzzy *Γ*-hyperideals of R*_1_ *and R*_2_ *, respectively, then μ* × *λ is a T-fuzzy *Γ*-hyperideal of R*_1_ × *R*_2_.

**Proof**

It is straightforward.

In [12], Dehkordi and Davvaz studied Noetherian and Artinian Γ-semihyperrings in crisp case. A collection *A* of subsets of a Γ-semihyperring *R* satisfies the ascending chain condition (or Acc) if there does not exist a properly ascending infinite chain *A*_1_ ⊂ *A*_2_ ⊂ ⋯ of subsets from *A*. Recall that a subset *B* ∈ *A* is a maximal element of *A* if there does not exist a subset in *A* that properly contains *B*. Similar to [18], in the following, we obtain some results related to fuzzy sets and Noetherian Γ-semihyperrings.

**Proposition 19 (see [12])**

Let *R* be a Γ-semihyperring. Then, the following conditions are equivalent:

1. *R satisfying the Acc condition on right (left) *Γ*-hyperideals, *
2. *every nonempty family of right (left) *Γ*-hyperideals has a maximal element, *
3. *every right (left) *Γ*-hyperideals is finitely generated.*

**Definition 20 (see [12])**

A Γ-semihyperring *R* is right (left) Noetherian if the equivalent conditions of the above proposition are satisfied. In the same way, we can define an Artinian Γ-semihyperring. Let *I* be a Γ-hyperideal of a Γ-semihyperring *R* and *I* be a Noetherian Γ-semihyperring. Then, *I* is called a Noetherian Γ-hyperideal of *R*.

**Example 21 (see [12])**

Let *A*_*n*_ = [*n*, *n*_*n*+1_] for every *n* ∈ *Z*, *R* = ⋃_*n*∈*Z*_ *A*_*n*_, and Γ = *Z*. Then, *R* is a Noetherian Γ-semihyperring with respect to the following hyperoperations:

(16) $$\begin{array}{c} x⊕y=A_{n+m},\;\;x\gamma y=A_{n\gamma m}, \end{array}$$

where *x* ∈ *A*_*n*_ and *y* ∈ *A*_*m*_.

**Theorem 22**

Let {*A*_*k*_ | *k* ∈ *N*} be a family of Γ-hyperideals of a Γ-semihyperring *R*, where *A*_1_⊃*A*_2_⊃*A*_3_⋯. Let *μ* be a fuzzy subset of *R* defined by

(17) $$\begin{array}{c} \mu \left(x\right)=\{\begin{array}{cc} \frac{k}{k+1} & if\;\;x\in A_{k}\setminus A_{k+1},\;\;k=0,1,2,\ldots \\ 1 & if\;\;x\in \overset{\infty }{\underset{k=0}{⋂}}A_{k}, \end{array} \end{array}$$

for all *x* ∈ *R*, where *A*_0_ stands for *R*. Let *T* be a* t*-norm with Im *μ*⊆Δ_*T*_. Then, *μ* is a *T*-fuzzy Γ-hyperideal of *R*.

**Proof**

Let *x*, *y* ∈ *R*. Suppose that *x* ∈ *A*_*k*_∖*A*_*k*+1_ and *y* ∈ *A*_*r*_∖*A*_*r*+1_ for *k* = 0,1, 2,… and *r* = 0,1, 2,…. Without loss of generality we may assume that *k* ≤ *r*. Then, obviously *y* ∈ *A*_*k*_. Since *A*_*k*_ is a Γ-hyperideal of *R*, it follows that *x* + *y*⊆*A*_*k*_ and *xαy*⊆*A*_*k*_ which imply that inf⁡_*z*∈*x*+*y*_ *μ*(*z*) ≥ *k*/(*k* + 1) = *T*(*μ*(*x*), *μ*(*y*)) and inf⁡_*z*∈*xαy*_ *μ*(*z*) ≥ *k*/(*k* + 1) = *μ*(*y*) for all *α* ∈ Γ. If *x* ∈ ⋂_*k*=0_^*∞*^ *A*_*k*_ and *y* ∈ ⋂_*k*=0_^*∞*^ *A*_*k*_, then *x* + *y*⊆⋂_*k*=0_^*∞*^ *A*_*k*_. Hence, inf⁡_*z*∈*x*+*y*_ *μ*(*z*) = 1 = *T*(*μ*(*x*), *μ*(*y*)) and inf⁡_*z*∈*xαy*_ *μ*(*z*) = 1 ≥ *μ*(*y*). If *x* ∉ ⋂_*k*=0_^*∞*^ *A*_*k*_ and *y* ∈ ⋂_*k*=0_^*∞*^ *A*_*k*_, then there exists *n* ∈ *N* such that *x* ∈ *A*_*n*_∖*A*_*n*+1_. It follows that *x* + *y*⊆*A*_*n*_ which implies that inf⁡_*z*∈*x*+*y*_ *μ*(*z*) ≥ *n*/(*n* + 1) = *T*(*μ*(*x*), *μ*(*y*)) and inf⁡_*z*∈*xαy*_ *μ*(*z*) ≥ *n*/(*n* + 1) = *μ*(*y*) for all *α* ∈ Γ. Finally, assume that *x* ∈ ⋂_*k*=0_^*∞*^ *A*_*k*_ and *y* ∉ ⋂_*k*=0_^*∞*^ *A*_*k*_, then *y* ∈ *A*_*n*_∖*A*_*n*+1_ for some *n* ∈ *N*. Therefore, *x* + *y*⊆*A*_*n*_, and thus inf⁡_*z*∈*x*+*y*_ *μ*(*z*) ≥ *n*/(*n* + 1) = *T*(*μ*(*x*), *μ*(*y*)) and inf⁡_*z*∈*xαy*_ *μ*(*z*) ≥ *n*/(*n* + 1) = *μ*(*y*), for all *α* ∈ Γ. Hence, *μ* is a *T*-fuzzy Γ-hyperideal of *R*.

**Theorem 23**

Let *R* be a Γ-semiring satisfying descending chain condition, let *μ* be a fuzzy subset of *R*, and let *T* be a *t*-norm with Im *μ*⊆Δ_*T*_. Let *μ* be a *T*-fuzzy Γ-hyperideal of *R*. If a sequence of elements of Im *μ* is strictly increasing, then *μ* has finite number of values.

**Proof**

Let {*t*_*k*_} be a strictly increasing sequence of elements of *Im*⁡*μ*. Then 0 ≤ *t*_1_ ≤ *t*_2_ ≤ ⋯≤1. Then, *U*(*μ*; *t*_*r*_) is an ideal of *M* for all *r* = 2,3,… Let *x* ∈ *U*(*μ*; *t*_*r*_). Then *μ*(*x*) ≥ *t*_*r*_ ≥ *t*_*r*−1_, and so *x* ∈ *U*(*μ*; *t*_*r*−1_). Hence *U*(*μ*; *t*_*r*_)⊆*U*(*μ*; *t*_*r*−1_). Since *t*_*r*−1_ ∈ *Im*⁡*μ*, there exists *x*_*r*−1_ ∈ *M* such that *μ*(*x*_*r*−1_) = *t*_*r*−1_. It follows that *x*_*r*−1_ ∈ *U*(*μ*; *t*_*r*−1_) but *x*_*r*−1_ ∉ *U*(*μ*; *t*_*r*_). Thus, *U*(*μ*; *t*_*r*_) ⊂ *U*(*μ*; *t*_*r*−1_) and so we obtain a strictly descending sequence *U*(*μ*; *t*_1_)⊃*U*(*μ*; *t*_2_)⊃*U*(*μ*; *t*_3_)⊃⋯ of Γ-hyperideals of *R* which is not terminating. This contradicts the assumption that *M* satisfies the descending chain condition. Consequently, *μ* has finite number of values.

**Theorem 24**

Let *R* be a Γ-semiring, *μ* be a fuzzy subset of *R*, and *T* be a *t*-norm with Im *μ*⊆Δ_*T*_. Then, the following conditions are equivalent:

1. *R* is a Noetherian Γ-semihyperring,
2. the set of values of any Γ-hyperideal of *R* is a well-ordered subset of [0,1].

**Proof**

(1)⇒(2): Let *μ* be a *T*-fuzzy Γ-hyperideal of *R*. Suppose that the set of values of *μ* is not a well-ordered subset of [0,1]. Then, there exists a strictly decreasing sequence {*t*_*k*_} such that *μ*(*x*_*k*_) = *t*_*k*_. It follows that *U*(*μ*; *t*_1_) ⊂ *U*(*μ*; *t*_2_) ⊂ ⋯ is a strictly ascending chain of Γ-hyperideals of *M*, where *U*(*μ*; *t*_*r*_) = {*x* ∈ *M* | *μ*(*x*) ≥ *t*_*r*_}, for every *r* = 1,2,…. This contradicts the assumption that *R* is a Noetherian Γ-semihyperring.

(2)⇒(1): Suppose that the condition (2) is satisfied and *R* is not a Noetherian Γ-hyperring. There exists a strictly ascending chain

(18) $$\begin{array}{c} A_{1}\subset A_{2}\subset A_{3}\subset \cdots \end{array}$$

of Γ-hyperideals of *R*. Note that *A* = ⋂_*k*=0_^*∞*^ *A*_*k*_ is a Γ-hyperideal of *R*. Define a fuzzy subset in *R* by

(19) $$\begin{array}{c} \mu \left(x\right)=\{\begin{array}{cc} \alpha & \text{if}\;\;x\notin A,\\ \frac{1}{r} & \text{where}\;\;r=\min\left\{k\in N\;|\;x\in A_{k}\right\}. \end{array} \end{array}$$

We claim that *μ* is a *T*-fuzzy Γ-hyperideal of *R*. Let *x*, *y* ∈ *R*. If *x* ∈ *A*_*k*_∖*A*_*k*−1_ and *y* ∈ *A*_*k*_∖*A*_*k*−1_, then *x* − *y* ⊂ *A*_*k*_ and *xαy* ⊂ *A*_*k*_. It follows that inf⁡_*z*∈*x*+*y*_ *μ*(*z*) = 1/*k* = *T*(*μ*(*x*), *μ*(*y*)) and inf⁡_*z*∈*xαy*_ *μ*(*z*) ≥ 1/*k* ≥ *μ*(*y*), for all *β* ∈ Γ. Suppose that *x* ∈ *A*_*k*_ and *y* ∈ *A*_*k*_∖*A*_*r*_ for all *r* < *k*. Since *A*_*k*_ is a Γ-hyperideal of *R*, it follows that *x* + *y* ⊂ *A*_*k*_. Hence, inf⁡_*z*∈*x*+*y*_ *μ*(*z*) ≥ 1/*k* ≥ 1/(*k* + 1) ≥ *μ*(*y*) and so inf⁡_*z*∈*x*+*y*_ *μ*(*z*) ≥ min⁡{*μ*(*x*), *μ*(*y*)} ≥ *T*(*μ*(*x*), *μ*(*y*)). Similarly, for the case *x* ∈ *A*_*k*_∖*A*_*r*_ and *y* ∈ *A*_*k*_, we have inf⁡_*z*∈*x*+*y*_ *μ*(*z*) ≥ min⁡{*μ*(*x*), *μ*(*y*)} ≥ *T*(*μ*(*x*), *μ*(*y*)) and inf⁡_*z*∈*xαy*_ *μ*(*z*) ≥ *μ*(*y*), for all *β* ∈ Γ. Thus, *μ* is a *T*-fuzzy Γ-hyperideal of *R*. Since the chain is not terminating, *μ* has a strictly descending sequence of values. This contradicts the assumption that the value set of any ideal is well-ordered. Therefore, *R* is a Noetherian Γ-semihyperring.

For a family {*μ*_*α*_ | *α* ∈ Λ} of fuzzy subsets in *R*, we define the join ∨_*α*∈Λ_ *μ*_*α*_ and the meet ∨_*α*∈Λ_ *μ*_*α*_ as follows:

(20) $$\begin{array}{c} \left(\underset{\alpha \in \Lambda }{⋁}\mu_{\alpha }\right)\left(x\right)=\sup\left\{\mu_{\alpha }\left(x\right)\;|\;\alpha \in \Lambda \right\},\\ \left(\underset{\alpha \in \Lambda }{⋀}\mu_{\alpha }\right)\left(x\right)=\inf\left\{\mu_{\alpha }\left(x\right)\;|\;\alpha \in \Lambda \right\}, \end{array}$$

for all *x* ∈ *R*, where Λ is any index set.

**Theorem 25**

The family of *T*-fuzzy Γ-hyperideals in *R* is a completely distributive lattice with respect to meet “∧” and join “∨”.

**Proof**

Since [0,1] is a completely distributive lattice with respect to the usual ordering in [0,1], it is sufficient to show that ∨_*α*∈Λ_ *μ*_*α*_ and ∧_*α*∈Λ_ *μ*_*α*_ are *T*-fuzzy Γ-hyperideals of *R* for family {*μ*_*α*_ | *α* ∈ Λ} of *T*-fuzzy Γ-hyperideals of *R*. For any *x*, *y* ∈ *R*, we have

(21) inf⁡z∈x+y(⋁α∈Λμα)(z)≥sup⁡{T(μα(x),μα(y)) ∣ α∈Λ}≥T(sup⁡⁡{μα(x) ∣ α∈Λ},sup⁡⁡{μα(y) ∣ α∈Λ})=T((⋁α∈Λμα)(x),(⋁α∈Λμα)(y)),inf⁡z∈x+y(⋀α∈Λμα)(z)≥inf⁡{T(μα(x),μα(y)) ∣ α∈Λ}=T((⋀α∈Λμα)(x),(⋀α∈Λμα)(y)).

Now, let *x*, *y* ∈ *M* and *β* ∈ Γ. Then

(22) inf⁡z∈xβy(⋁α∈Λμα)(z)≥sup⁡{μα(y) ∣ α∈Λ}=(⋁α∈Λμα)(y),

(23) inf⁡z∈xβy(⋀α∈Λμα)(z)≥inf⁡{μα(y) ∣ α∈Λ}=(⋀α∈Λμα)(y).

Hence, ∨_*α*∈Λ_ *μ*_*α*_ and ∧_*α*∈Λ_ *μ*_*α*_ are *T*-fuzzy Γ-hyperideals of *R*. This completes the proof.
